# Multiple comorbidities in patients with long-lasting chronic spontaneous urticaria^☆^

**DOI:** 10.1016/j.abd.2022.03.004

**Published:** 2022-10-29

**Authors:** Rosana Câmara Agondi, Paula Natassya Argôlo, Mariana Mousinho-Fernandes, Bruna Gehlen, Jorge Kalil, Antonio Abílio Motta

**Affiliations:** a Clinical Immunology and Allergy Division, Faculty of Medicine, Universidade de São Paulo, São Paulo, SP, Brazil; b Laboratory of Immunology (LIM19), Instituto do Coração (InCor), Faculty of Medicine, Universidade de São Paulo, São Paulo, SP, Brazil

Dear Editor,

Chronic Spontaneous Urticaria (CSU) is a mast cell-driven skin disease, and mast cell degranulation is triggered by the activation of several receptors on its surface. IgE-FcεRI complex appears to be involved in the autoimmune etiology of CSU, through the presence of IgG anti-FcεRI, anti-IgE or IgE against autoallergens, but many other receptors can induce mast cell degranulation, as such as Mas-Related G-Protein-Coupled Receptor X2 (MRGPRX2).^1^, ^2^, ^3^

Several types of comorbidities are associated with CSU and often lead it to a worse prognosis. One of the most common comorbidities is an autoimmune disease. CSU, itself considered an autoimmune disease, is mainly associated with autoimmune thyroid disease, and the high prevalence of these autoimmune diseases in patients with CSU supports that hypothesis. Other comorbidities often associated with CSU are psychological disorders; infectious diseases, including viruses, bacteria, and parasites; as well as with metabolic syndrome (MetS).^3^, ^4^, ^5^, ^6^, ^7^

In this retrospective and observational study, we evaluated the association between long-lasting CSU and the occurrence of multimorbidities in patients followed up (one year or more) in a tertiary Center – UCARE, between January 2019 and December 2020.

Patients were classified according to the duration of the CSU: 1 to 2 years; 3 to 5 years; 6 to 10 years; and 11 or more years. Groups were evaluated for demographic data, angioedema, and refractoriness to antihistamines. Comorbidities assessed were gastrointestinal disorders, obesity, systemic arterial hypertension, diabetes mellitus, dyslipidemia, autoimmune thyroid diseases, respiratory diseases, and psychological disorders.

MetS comprehends a constellation of cardiovascular disease risk factors, which includes glucose intolerance, dyslipidemia, hypertension, and central abdominal obesity.^8^ At least three of those four components have to being fulfilled for MetS diagnosis in CSU patients.

We included 173 patients with CSU, 86.1% were women with a mean age of 49.8 years. Angioedema was reported by 112 (64.7%) patients, *nonsteroidal anti-Inflammatory drugs*-exacerbated CSU was observed in 71 (41.0%), and 45 (26.0%) patients were refractory to antihistamines (four times daily). When patients with CSU were classified according to the duration of the disease, 28 (16.2%) had 1 or 2 years ; 51 (29.5%), 3 to 5 years ; 37 (21.4%) between 6 and 10 years, and 57 (32.7%) had 11 years or more of disease. General characteristics can be observed in Table 1.

**Table 1:** Demographic and clinical data of patients with CSU according to the duration of disease.

General characteristics	Duration of CSU				p
	1 and 2 years	3 and 5 years	6 and 10 years	11 or more years	
Gender: female	75.0	86.3	89.2	89.5	NS
Current age (year; mean, SD)	47.9	46.9	50.3	52.8	NS
Age at onset of CSU (y; mean, SD)	46.2	43.1	42.5	33.2	<0.001
Duration of CSU	1.8	4.1	7.8	19.5	NA
CIndU (%)	32.1	33.3	43.2	31.6	NS
Angioedema (%)	46.4	70.6	62.6	70.2	NS
NSAID exacerbated CSU (%)	10.7	43.1	43.2	52.6	0.002
Refractoriness to antistamines (%)	14.3	23.5	24.3	35.1	NS

CSU, Chronic Spontaneous Urticaria; SD, Standard Deviation; NA, not applicable; CIndU, Chronic Inducible Urticaria; NSAID, Nonsteroidal Anti-Inflammatory Drugs, NS, Non-significant

The Kruskal-Wallis test for current age and age at onset of CSU.

χ^2^ analysis for gender, CIndU, angioedema, NSAID exacerbated CU, and refractoriness to antihistamine.

There was no statistical difference in relation to the current age of the patients in those different groups (according to the duration of the CSU), although, the age at onset of CSU was lower for patients with long-lasting CSU, and these patients had more often multimorbidities, including the components of MetS (p < 0.05).

Eight comorbidities were assessed in this study, the frequency of them in the same patient ranged from 0 to 6 comorbidities (Fig. 1). The most frequent comorbidities were respiratory disorder (rhinitis and/or asthma) in 82 (47.4%), followed by high blood pressure, in 54 (31.2%), and dyslipidemia, in 38 (22%) patients. There was a correlation between the duration of CSU and the frequency of comorbidities, *r*^2^ = 0.043, p = 0.007 (Fig. 2).

**Figure 1:**
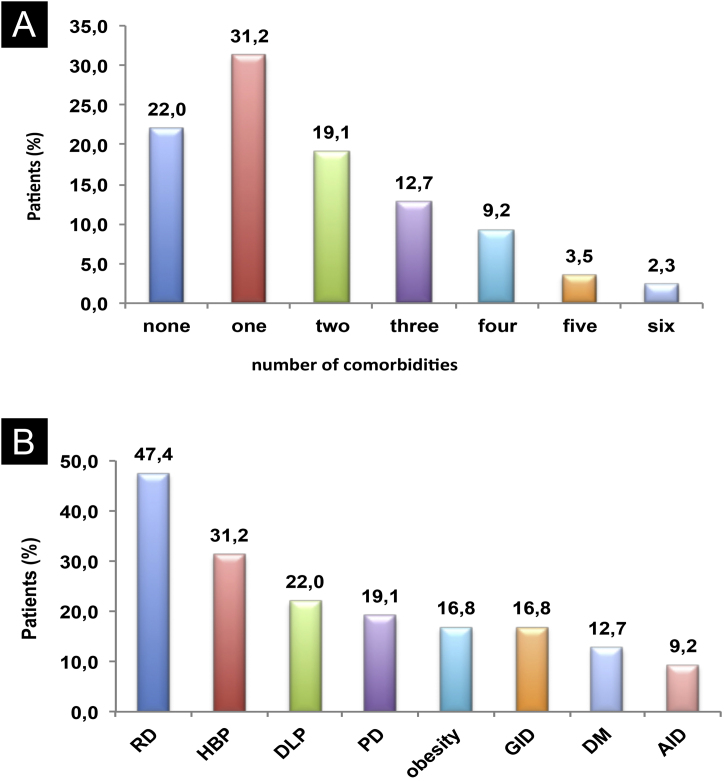
Comorbidities: (A) Co-prevalence of comorbidities (0 to 6) in the same patient; (B) Frequency of each comorbidity in patients with CSU. RD, Respiratory Disorders; HBP, High Blood Pressure; DLP, Dyslipidemia; PD, Psychiatric Disorders; GID, Gastrointestinal Disorders; DM, Diabetes Mellitus; AID, Autoimmune Diseases.

**Figure 2:**
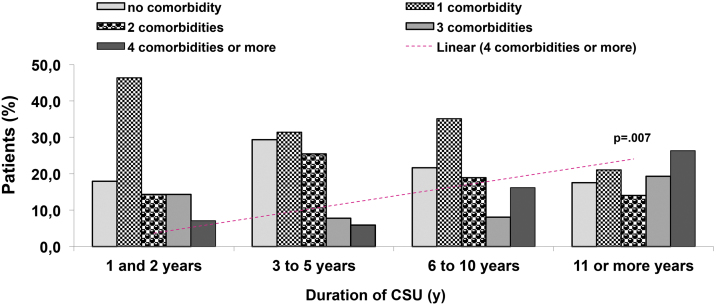
Frequency of comorbidities according to the duration of CSU; y, year; *χ*^2^ test, p = 0.007.

Patients with CSU refractory to antihistamine had a higher frequency of angioedema, gastrointestinal disorders, and obesity, compared to those patients responsive to antihistamine (77.8% versus 60.2%, p = 0.045; 28.9% versus 13.3%, p = 0.017; and 26.7% versus 16.4%, p = 0.039, respectively).

In the present study, 16 (9.2%) patients had CSU-associated autoimmune diseases, a frequency higher than that observed in the general population (around 5%).^6^ Of the 16 patients, autoimmune thyroid disease represented 87.5% of them. However, we did not find an increase in its frequency as CSU lasted longer or with disease severity. One explanation would be that the mean age in the studied groups, according to the duration of CSU, was similar (mean of 49.8 years), reinforcing that the frequency of autoimmune diseases increases with age.^4^

Multimorbidity is associated with a poor quality of life; patients are at higher risk of severe clinical outcomes. CSU is nowadays considered a low-grade inflammatory systemic disease. The proposed pathomechanism would be the constant or continuous activation of mast cells observed in patients with uncontrolled CSU. In addition to the autoimmune mechanism described for mast cell activation in patients with CSU, several other factors maintain or worsen the mast cell activation.^9^, ^10^ In the present study, three components or more of MetS were observed more frequently in patients with six years or more of CSU, compared with those patients with one to five years of disease (15.9% versus 1.3%, p < 0.001). The most common component of MetS was high blood pressure (31.2% of 173 patients), and around 10% of 173 patients had at least three of them.

In conclusion, this study showed that patients with early-onset and long-lasting CSU had more often comorbidities including three or more components of MetS. The present study has shown that almost half of the participants had at least one component of MetS. Three or more components of MetS had a statistically significant higher frequency in patients with CSU lasting six or more years. Long-lasting CSU, and probably, uncontrolled disease, could evolve with multimorbidities, suggesting that early complete control of CSU would be essential to prevent this outcome. These results need further prospective research to highlight the importance of CSU as a low-grade inflammatory disease. The present results suggest an association between long-lasting CSU and MetS, but case-control trial studies should be essential to confirm the our conclusions, further including a larger number of participants.

## Financial support

None declared.

## Authors' contributions

Rosana Câmara Agondi: Statistical analysis; approval of the final version of the manuscript; design and planning of the study; drafting and editing of the manuscript; collection, analysis, and interpretation of data; effective participation in research orientation; intellectual participation in the propaedeutic and/or therapeutic conduct of the studied cases; critical review of the literature; critical review of the manuscript.

Paula Natassya Argolo: Statistical analysis; approval of the final version of the manuscript; design and planning of the study; drafting and editing of the manuscript; collection, analysis, and interpretation of data; effective participation in research orientation; intellectual participation in the propaedeutic and/or therapeutic conduct of the studied cases; critical review of the literature; critical review of the manuscript.

Mariana Mousinho-Fernandes: Statistical analysis; approval of the final version of the manuscript; design and planning of the study; drafting and editing of the manuscript; collection, analysis, and interpretation of data; effective participation in research orientation; intellectual participation in the propaedeutic and/or therapeutic conduct of the studied cases; critical review of the literature; critical review of the manuscript.

Bruna Gehlen: Statistical analysis; approval of the final version of the manuscript; design and planning of the study; drafting and editing of the manuscript; collection, analysis, and interpretation of data; effective participation in research orientation; intellectual participation in the propaedeutic and/or therapeutic conduct of the studied cases; critical review of the literature; critical review of the manuscript.

Jorge Kalil: Statistical analysis; approval of the final version of the manuscript; design and planning of the study; drafting and editing of the manuscript; collection, analysis, and interpretation of data; effective participation in research orientation; intellectual participation in the propaedeutic and/or therapeutic conduct of the studied cases; critical review of the literature; critical review of the manuscript.

Antonio Abílio Motta: Statistical analysis; approval of the final version of the manuscript; design and planning of the study; drafting and editing of the manuscript; collection, analysis, and interpretation of data; effective participation in research orientation; intellectual participation in the propaedeutic and/or therapeutic conduct of the studied cases; critical review of the literature; critical review of the manuscript.

## Conflicts of interest

None declared.
